# Childhood social isolation causes anxiety-like behaviors via the damage of blood-brain barrier in amygdala in female mice

**DOI:** 10.3389/fcell.2022.943067

**Published:** 2022-08-16

**Authors:** Xiao Wu, Zengbo Ding, Tengteng Fan, Ke Wang, Suxia Li, Jing Zhao, Weili Zhu

**Affiliations:** ^1^ School of Basic Medical Sciences, Peking University, Beijing, China; ^2^ National Institute on Drug Dependence and Beijing Key Laboratory of Drug Dependence, Peking University, Beijing, China; ^3^ Peking University Sixth Hospital, Peking University Institute of Mental Health, NHC Key Laboratory of Mental Health (Peking University), National Clinical Research Center for Mental Disorders (Peking University Sixth Hospital), Beijing, China; ^4^ Department of Neurology, Minhang Hospital, Fudan University, Shanghai, China

**Keywords:** social isolation, childhood, anxiety, amygdala, blood-brain barrier, Claudin-5, neuroinflammation

## Abstract

Social interaction plays an essential role in species survival for socialized animals. Previous studies have shown that a lack of social interaction such as social isolation, especially in the early-life phase, increases the risk of developing mental diseases in adulthood. Chronic social stress alters blood-brain barrier (BBB) integrity and increases peripheral cytokines to infiltrate the brain, which is linked to the development of depressive-like behaviors in mice, suggesting that BBB function is crucial in environmental stimuli-driven mood disorders via increased neuroinflammation in the brain. However, the precise mechanisms of inflammation and BBB integrity underlying the behavioral profiles induced by social isolation remain poorly understood. Here we showed that chronic childhood social isolation from post-weaning for consecutive 8 weeks in female but not male C57BL/6J mice induces anxiety-like behaviors. The levels of peripheral inflammatory cytokines including interleukin (IL)-1β, IL-6, and tumor necrosis factor (TNF)-α in the plasma of socially isolated female mice were increased. Importantly, we found decreased expression of the endothelial cell tight junction protein Claudin-5, increased BBB breakdown and microglial activation in the amygdala of isolated but not group-housed female mice. Moreover, the neuronal activity in the amygdala was increased as evidenced by c-fos positive cells, and the levels of IL-1β in the amygdala, a critical brain region for regulating social processing and interaction, were also higher in female mice exposed to social isolation. Finally, down-regulation of Claudin-5 induced anxiety-like behaviors in group-housed females and overexpression of Claudin-5 with adeno-associated virus in the amygdala to restore BBB integrity decreased subsequent anxiety-like behaviors. Together, these findings suggest that chronic childhood social isolation impaired BBB permeability and caused neuroinflammation in the amygdala by recruiting peripheral cytokines into the brain and activating microglia, consequently triggering the development of anxiety-like behaviors in female mice.

## Introduction

Social behavior, like eating and sleeping, is an essential function for both socialized animals and human beings survival and development (Baumeister and Leary, 1995; Cacioppo et al., 2014). Lack of social interaction, as a psychological stress factor, lead to mental disorders such as anxiety and depression (Cacioppo et al., 2014; Chen et al., 2016; Endo et al., 2018; Xia and Li, 2018; Campagne, 2019; Fosnocht et al., 2019; Pais et al., 2019; Taheri Zadeh et al., 2021). Moreover, comparing to men, women are more prone to mental health conditions after experiencing social isolation (Sepúlveda-Loyola et al., 2020; Smith et al., 2020; Taheri Zadeh et al., 2021). Specifically, most anxiety disorders affect almost twice as many women as men (Craske and Stein, 2016). However, the majority of the currently used anxiety models was conducted in males and resulted in insufficient evidence on the pathogenesis of anxiety-related psychiatric disorders, leading to one-third of social anxiety disorder patients are treatment resistant due to the low response to conventional treatments (Craske et al., 2017; Malhi and Mann, 2018; Slavich and Sacher, 2019; Smith et al., 2020; Fruehwirth et al., 2021; Dion-Albert et al., 2022). Childhood and adolescence are susceptible periods for social isolation, during which the brain undergoes enhanced synaptic plasticity related to social function (Petanjek et al., 2011; Orben et al., 2020). Previous studies have shown that social isolation, especially in the early-life phase, increases the risk of developing mental diseases in adulthood (Rivera-Irizarry et al., 2020). Moreover, social isolated male mice exhibit heightened aggression, while isolated females exhibit social withdrawal, revealing a sex-specific responses to isolation stress (Tan et al., 2021). Therefore, it is an urgent need to determine the mechanism involed in the diverse effects of childhood social isolation on behavioral consequences in both sexes.

Exposure to inflammation is associated with several neuropsychiatric disorders, including depression and anxiety (Maes et al., 1995; Smith et al., 2020). Among the studies on the pathogenesis of mental disorders induced by social isolation, one of the most commonly demonstrated stress responses induced by social isolation is systemic inflammatory response (Krügel et al., 2014; Corsi-Zuelli et al., 2018; Almeida et al., 2021). The blood-brain barrier (BBB) is a bridge of humoral pathways between peripheral inflammation and neuroinflammation. Recently, the critical role of the BBB in the pathogenesis of mental disorders has received a particular attention on sex-specific characteristics (Menard et al., 2017; Dion-Albert et al., 2022). For example, male mice experiencing social defeat stress demonstrated BBB damage and decreased tight junction protein Claudin-5 in the nucleus accumbens (NAc), while female mice showed a damaged BBB in the medial prefrontal cortex (mPFC) (Menard et al., 2017; Dion-Albert et al., 2022). These findings highlighted the significance of further determining the role of region-specific dysregulation of BBB integrity in diverse behavioral phenotypes in males and females suffered early-life social isolation.

This study aims to clarify the mechanisms underlying sex-specific influence of behavioral outcomes induced by chronic social isolation. We exposed mice from post-weaning for 8 weeks of social isolation and measured anxiety-, depressive-like behaviors, social interaction and cognitive functions. In addition, peripheral inflammation, neuroinflammation and BBB function were measured. Finally, we regulated the tight junction protein Claudin-5 using virus-mediated manipulation to reveal the role of the BBB in behavioral deficits induced by social isolation.

## Materials and methods

### Animals

Both male and female C57BL/6J mice (Peking University Health Science Center) were used in this study and maintained in standard temperature (22 ± 2°C), humidity (50% ± 10%), and 12 h circadian rhythm cycle (8:00–20:00 is the light cycle) with free food and water access. Mice were 3 weeks old at the start of the experiments (weighing 7–9 g). Behavioral tests were carried out during the dark cycle. All mouse experiments met the ethical requirements of experimental animals and complied with the regulations issued by the state and the Animal Use and Protection Committee of the Peking University Health Science Center (Approval No. LA2019067).

### Childhood social isolation stress

On post-natal day 21 (PND21), mice were housed in isolation (1 mouse per cage) or groups (4–6 mice per cage). Mice were placed in the same rooms, but baffles were arranged between adjacent cages for isolated mice. Isolated mice can hear and smell other mice but cannot see and interact physically with other mice. According to previous study, social isolation procedure was maintained for 8 weeks (Gan et al., 2014).

### Novelty suppressed feeding test

The novelty suppressed feeding test (NSFT) was performed in an opaque acrylic plate box with an open top (42 cm × 42 cm × 42 cm). Mice were tested after 24 h of food deprivation. Four food pellets from home cage were placed in the central area of the test box. Mice were gently put into the test box from one corner. The latency to start eating food pellets was used to evaluate the anxiety-like behaviors. During the test, mouse was allowed to explore freely in the box for 10 min, and the longest latency was recorded as 600 s (Linge et al., 2013).

### Elevated plus maze test

The elevated plus maze (EPM) apparatus consists of two open arms (30 cm × 5 cm), two closed arms (30 cm × 5 cm, height 23 cm), and a central area (5 cm × 5 cm); the open arms were elevated 75 cm from the ground. During testing, mice were gently placed in the central area with their head facing the open arm, and the test lasted for 6 min. The EPM test is a widely accepted approach to reflect the behavioral changes following psychosocial stress. The time that mice stayed in open arms and the numbers entered open arms were recorded as indicators to evaluate anxiety-like behaviors (Pi et al., 2020).

### Open field test

The open field test (OFT) was performed in an opaque acrylic plate box with an open top (42 cm × 42 cm × 42 cm). Mice were placed in the center of the apparatus in the beginning of the test, and were allowed to explore freely for 5 min. Total travel distance (cm) was recorded as the measure of spontaneous locomotion. The time of mice in the central area (24 cm × 24 cm) was recorded as a measure of anxiety-like behaviors. At the end of the test, mice were put back to their home cages. During the test interval of two mice, the test apparatus was wiped with 75% alcohol to remove the residual odor and excreta (Linge et al., 2013).

### Sucrose preference test

The sucrose preference test (SPT) is a useful parameter of anhedonia simulating a core symptom of depression in humans based on a two-bottle free-choice paradigm in rodents. Briefly, the sucrose preference test consists of three stages: 1) Adaptation stage: 1% sucrose solution was prepared with daily drinking water of mice, 1% sucrose and drinking water were put into two bottles of the same size and appearance, respectively, and then two bottles were placed on the cages of mice. Mice can freely choose to drink each liquid from the two bottles for 48 h, and the positions of the two bottles were exchanged at the 24th hour. 2) Fasting stage: mice were deprived of water and food for 24 h before testing. 3) Sucrose preference test: 1% sucrose bottles and drinking water bottles were weighed and carefully placed on the cage simultaneously. Mice can freely choose to drink either liquid from the two bottles for 24 h. The positions of the two bottles were exchanged at the 12th h. After testing, two bottles were weighed again, and the intakes of sucrose and drinking water within 24 h were calculated. The sucrose preference value was used as the indicator of anhedonia (Liu et al., 2018). Sucrose preference (%) = 1% sucrose intake/(1% sucrose intake + water intake) × 100%.

### Forced swim test

The forced swim test (FST) was conducted in a plexiglass cylinder with a diameter of 18 cm and a height of 27 cm. The water depth was 15 cm, and the water temperature was kept at 25 ± 1°C. When testing, mouse cannot be supported by their limbs or tail. Baffles were placed between adjacent cylinders to prevent mice from learning from each other. The water in the cylinder was replaced every three mice to avoid the interference of excreta. The forced swim test was divided into two stages:1) Adaptation stage: 1 day before the test, mice were forced to swim for 15 min. 2) Forced swim test: 24 h later, mice were subjected to the swimming test for 6 min, and the floating immobility time of the last 4 min after recording was used as an indicator of depressive-like and despair behavior (Snyder et al., 2011).

### Tail suspension test

For the tail suspension test (TST), mouse was hung up by the tape (3/4 of the distance from the bottom of tail) and were suspended about 35 cm away from the ground after hanging. A total of 6 min of testing was carried out and the time that mice spent immobility in the last 4 min of each 6-min test was recorded as an indicator of despair behavior (Stukalin et al., 2020).

### Social interaction test

The social interaction (SI) test equipment consists with an open top (42 cm × 42 cm × 42 cm), and the social interaction area was a 12 cm × 24 cm rectangle on one side within a 7 cm × 11 cm × 30 cm transparent plastic box. This box separated the test mouse from the other mouse used for interaction. The top of this box was open, and there were some holes at its walls so that the test mouse could smell the odor of the mouse used for interaction (Tian et al., 2018). The test was divided into two sessions, each session was 2.5 min, and the interval between the two sessions was 1 min. In the first session, there were no mice in the transparent plastic box, and the test mouse was put into the test device from the corner away from the interaction area. Then, the test mouse was allowed to explore freely for 2.5 min. In the second 2.5 min session, an unfamiliar mouse was placed into the transparent plastic box. The test mouse was again put into the test device from the corner away from the interaction area again. The time that mice spent in interaction zone of two sessions were recorded, respectively. The social behavior of mice was evaluated by the SI index calculated by the ratio of time that mice spent in the interaction area in the second session to the time that mice spent in the interaction area in the first sessions (Iñiguez et al., 2014).

### Novel object recognition test

The novel object recognition test (NORT) was performed in a 40 cm × 27 cm × 18 cm box. The test was divided into two sessions, each session was 3 min, and the interval between two sessions was 15 min. In the first session, mice were placed in the test device from the corner. There were two same objects A in the center of the test box. In the second session, there were two objects: familiar object A and novel object B. A and B were different in character, material, and color. Mice were placed in the test device from its corner, and the time of mice exploring A and B was recorded, respectively. The cognitive function of mice was evaluated with the novel object recognition index, which is determined by a ratio of the time of exploring object B to (the time of exploring object A + the time of exploring object B) (Janczura et al., 2013).

### Western blot

Mice were sacrificed, and brain tissues were frozen in n-hexane cooled with dry ice and ethanol (−65°C) and were stored in a −80°C refrigerator until use. The positions of the mPFC, NAc, amygdala (Amy), hypothalamus (Hypo) and hippocampus (Hip) were determined according to the second edition of Mouse Brain in Stereotaxic Coordinates and brain tissues were removed with a freezing microtome (Leica, CM1900). The collected tissues were homogenized in RIPA lysis buffer (Applygen, C1053) supplemented with protease inhibitors cocktail (Applygen, P1266) and phosphatase inhibitors cocktail (Applygen, P1264) and were homogenized by bead rotor homogenizer (Omini). After centrifugation (13,000 rpm for 15 min at 4°C), the supernatant was collected, and protein concentration was quantified with BCA Protein Assay Kits (Applygen, P1511). Protein samples were adjusted to an equal concentration and were mixed with 5 × loading buffer (Applygen, B1012) and heated at 100°C for 5 min to be denaturalized. Protein samples were separated by SDS-PAGE electrophoresis (Meilun 12% gels, MA0260) at 120 V (for about 1 h), subsequently were electrotransferred onto polyvinylidene difluoride membranes (Millipore) via wet transfer (250 mA at 4°C for 1 h). Protein membranes were blocked using 5% defatted milk powder in TBST buffer for 1 h at room temperature. The membranes were incubated overnight with primary antibodies against Claudin-5 (1:1,000, Invitrogen, rabbit, 34–1,600), IL-1β (1:1,000, Abcam, rabbit, Ab234437), TNF-α (1:500, Abcam, rabbit, Ab183218), IL-6 (1:1,000, Abcam, rabbit, Ab229381), and β-actin (1:3,000, ZSGB-BIO, mouse, TA-09) overnight at 4°C. The membranes were washed using TBST for 3 × 10 min and were incubated with appropriate secondary antibody conjugated to horseradish peroxidase (1:2,000, ZSGB-BIO, goat anti-rabbit, ZB-2301; 1:2,000, ZSGB-BIO, goat anti-mouse, ZB-2305) for 1 h. Chemiluminescence was performed with a hypersensitive ECL Chemiluminescence kit (Millipore) and visualized using a chemiluminescence Imager (GelDoc XR). β-actin was set as a loading control, and the signal of immunoblot was analyzed by Image J software.

### Enzyme-linked immunosorbent assay

Mice were anesthetized with 5% chloral hydrate, and blood was collected from the inferior vena cava. After anticoagulation by EDTA-K_2_ EP tube, blood sample was centrifuged (2,500 rpm for 15 min). The supernatant was the blood plasma and was stored at −20°C until use. According to mouse IL-1β, IL-6, TNF-α, S100 Enzyme-linked immunosorbent assay (ELISA) Kit instructions (Meimian, MM-0040M2, MM-0163M2, MM-0132M2, MM-0038M1), the kit was placed at room temperature for 30 min in advance, and 50 μl of different concentrations of standard samples, 40 μl of sample diluent, and 10 μl of sample + 40 μl of sample diluent were added to the standard wells, blank wells, and sample wells respectively. 100 μl of enzyme labeling reagent was added to each well (excluding blank wells), and the plate was sealed with a sealing film. After incubation at 37°C for 1 h, each well was washed with washing solution 5 × 30 s, and then patted dry. Each well was added 50 μl of chromogenic reagent and 50 μl of chromogenic reagent B. After incubation at 37°C for 15 min in the dark, 50 μl of stop solution was added to each well. Absorbance was detected in microplate photometer at 450 nm (Multiskan^TM^FC, Thermo Fisher Scientific). Then the absorbance was calculated as concentration from the working curve. Furthermore, the value was normalized with the average of the control group for subsequent statistical analysis.

### Quantitative real-time PCR

Total RNA was extracted from mouse amygdalar tissues with Trizol reagent (Invitrogen) and was quantified by AceQ Universal SYBR qPCR Master Mix Kit (Vazyme, Q511) under the following conditions: (95°C for 10 min; 40 cycles of 95°C for 15 s, 65°C for 1 min, 95°C for 15 s; 60°C for 1 min, 95°C for 1 s). Primers sequences (Sangon Biotech) for amplification were follows CLDN5 (Forward 5′-TTT​CTT​CTA​TGC​GCA​GTT​GG-3′, Reverse GCA​GTT​TGG​TGC​CTA​CTT​CA-3′), GAPDH (Forward 5′-AAC​TTT​GGC​ATT​GTG​GAA​GG-3′, Reverse 5′-GGA​TGC​AGG​GAT​GAT​GTT​CT-3′).

### Immunofluorescence

We anesthetized mice with 5% chloral hydrate, perfused with 0.1 M PBS and 4% paraformaldehyde (PFA) successively. Subsequently, mice brains were removed and postfixed in 4% PFA for 24 h, dehydrated in 30% sucrose three times, and frozen by n-hexane on dry ice. The brains were then sliced into 20 μm thick coronal sections on the freezing microtome (Leica, CM1900). All of the sections were soaked in blocking solution (5% bovine serum albumin, Biofroxx, 9048-46-8, and 0.3% Triton X-100 (Sigma-Aldrich) in 0.1 M PBS) for 1 h at 37°C. Sections were incubated with primary antibodies in a blocking solution for 36 h at 4°C (c-fos, 1:1,000, Cell Signaling Technology, Rabbit, 2,250; Iba-1, 1:1,000, Abcam, Rabbit, ab178846). After washing 3 × 10 min in 0.1 M PBS, sections were incubated with goat anti-rabbit alexa Fluor 488 secondary antibodies (1:1,000, abcam, Ab150077) for 3 h at room temperature. The sections were rewashed 3 × 10 min with 0.1 M PBS. Images of amygdala were acquired with fluorescence microscope (Olympus VS120) and analyzed by Image J software referring to previous report (Zhang et al., 2016).

### Blood cell count

Mice were anesthetized with 5% chloral hydrate. The blood was collected from the inferior vena cava and were quickly placed into the EDTA-K_2_ anticoagulant tube (IDXX Vet Collect™). Within 6 h after collecting, the blood cell analysis was performed with a five-classification automatic blood cell analyzer (IDEXX procyte DX™). Peripheral inflammation markers including leukocytes counts, neutrophils counts, neutrophils percentage, neutrophil/lymphocyte ratio (NLR), monocytes counts, monocytes percentage, lymphocytes counts and lymphocytes percentage were recorded.

### Stereotaxic injection

Mice were anesthetized with isoflurane, and the head of mice were fixed in stereotaxic instrument (RWD Life Science). AAV particles were injected bilaterally into the amygdala of the mouse brain (anterior/posterior, −1.2 mm; medial/lateral, ±3.1 mm; dorsal/ventral, −5.2 mm) using hamilton syringes at a rate of 2 nl/min, needles were left in the place for additional 10 min to allow the virus to diffuse from the injection site (Manzano Nieves et al., 2020).

### AAV of intervening protein expression

The AAVs used in this experiment were purchased from Sunbio (Shanghai, China). Short-hairpin RNA (shRNA) AAV: AAV9-CAG-*Cldn5*-shRNA-mCherry and its negative control virus AAV9-CAG-shRNA-mCherry were used for down-regulation of *Cldn5*, and their titers were 5.03 × 10^12^ and 5.58 × 10^12^ vg/ml, respectively. The virus construction was referred to previous report (Menard et al., 2017). The overexpression of *Cldn5* was performed by using overexpression adeno-associated virus: AAV9-CAG-*Cldn5*-mCherry and its negative control virus AAV9-CAG-mCherry, with titers of 7.31 × 10^12^ and 2.58 × 10^13^ vg/ml, respectively. The negative control virus was diluted 3.5 times with 0.1 M PBS before use. After 4 weeks of AAV stable expression, the level of Claudin-5 protein was detected by Western blot.

### Transmission electron microscope

Mice were anesthetized with 5% chloral hydrate and perfused with 0.1 M PB and 3% glutaraldehyde. The amygdala tissues were dissected into 1 mm^3^ blocks, and postfixed in 3% glutaraldehyde overnight, dehydrated in 30% sucrose for three times. Next, the tissue blocks were fixed in 1% osmium tetroxide for 2 h at 4°C and were dehydrated using graded acetone and then immersed in acetone/epoxy resin (1:1) for 2 h, acetone/epoxy resin (1:2) for 4 h. The blocks were embedded by epoxy resin at 37°C for 24 h, 45°C for 24 h, 60°C for 24 h. The embedded blocks were sliced into ultrathin sections (50–70 nm) using an ultramicrotome (Leica, Germany, EM UC7). Finally, the micrographs were captured under the transmission electron microscope (Leica, Germany, JEM-1400PLUS).

### Statistical analysis

All statistical analyses were performed with Graphpad Prism 8.0 software. D’Agostino & Pearson normality was used to test the normal distribution, and then Brown-Forsythe was used to test the homogeneity of variance. If the data conforms to the normal distribution and homogeneity of variance, the two-tailed unpaired T-tests were used to analyze the univariate data in experiments with two groups. If the variance of univariate data was unequal, the Mann-Whitney test was used to analyze. Two-way ANOVA was used for bivariate data, and then a post hoc comparison was carried out. Bonferroni’s test was used to test the difference between groups. *p* < 0.05 was used as the standard to measure the significant difference, and the results were expressed as mean ± SEM.

## Results

### Childhood social isolation induced anxiety-like behaviors in female but not male mice

**FIGURE 1:**
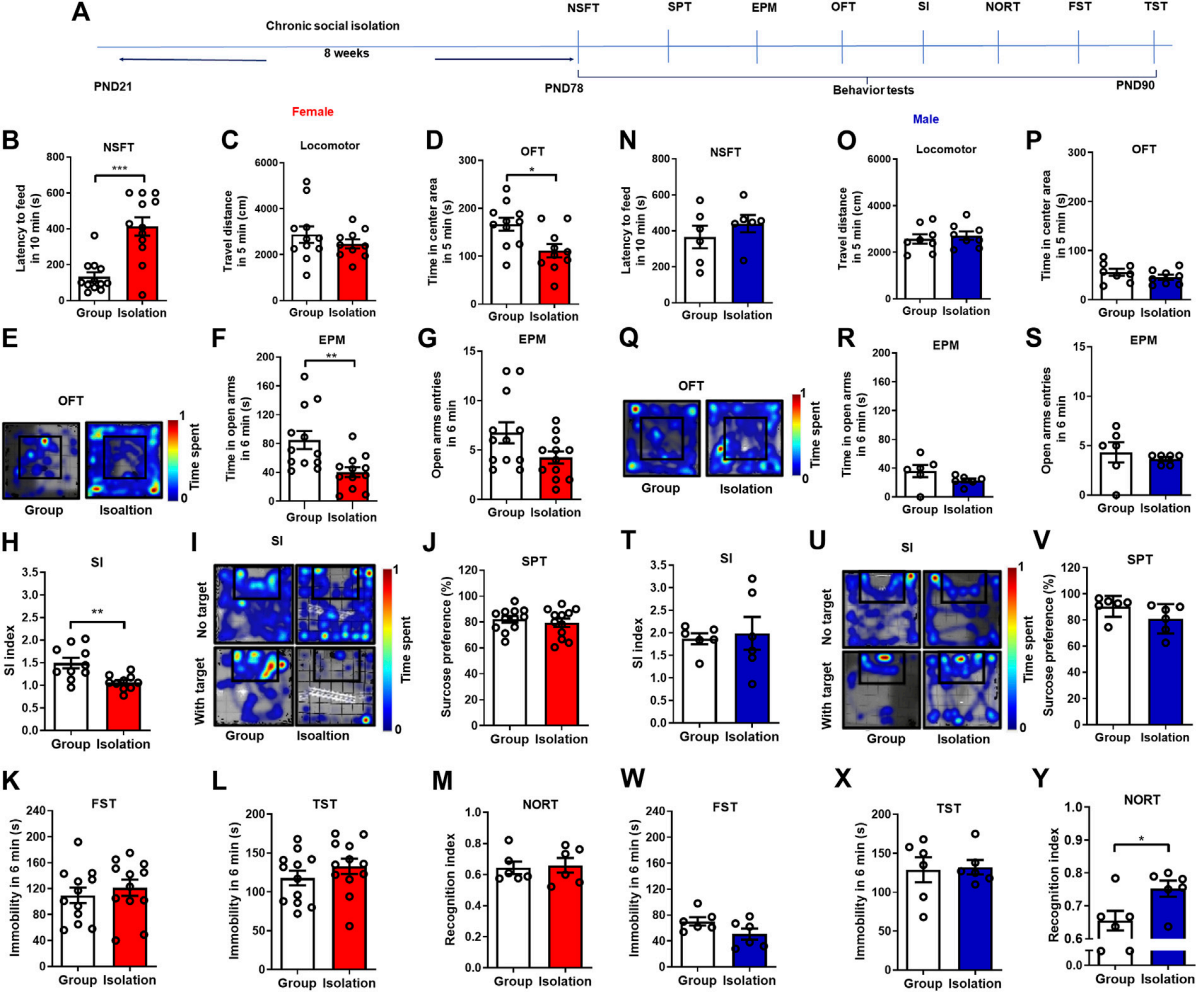
Chronic social isolation induced anxiety-like behaviors in female but not male mice. **(A)** Timeline of the experimental procedure. **(B)** The novelty suppressed feeding test in female mice (red bars, *n* = 10–12). **(C)** The locomotor test. **(D,E)** Time spent in the center area and representative heat maps during open field test. **(F,G)** Time and entries in the open arms in the elevated plus maze test. **(H,I)** The social interaction test and representative heat maps. **(J)** The sucrose preference test. **(K)** The forced swim test. **(L)** The tail suspension test. **(M)** The novel object recognition test. **(N)** The novelty suppressed feeding test in male mice (blue bars, *n* = 6–8). **(O)** The locomotor test. **(P,Q)** Time spent in the center area and representative heat maps during open field test. **(R,S)** Time and entries in the open arms in the elevated plus maze test. **(T,U)** The social interaction test and representative heat maps. **(V)** The sucrose preference test. **(W)** The forced swim test. **(X)** The tail suspension test. **(Y)** The novel object recognition test. Data were mean ± SEM, *p < 0.05, **p < 0.01, ***p < 0.001.

Although various models of chronic childhood social isolation have been developed in mice for several years, the results obtained from different studies are not entirely consistent due to the animal species, sex, behavioral measurements, and procedure details (Chen et al., 2016; Huang et al., 2017; Yamamuro et al., 2020; Salihu et al., 2021). We first examined the effects of 8-week social isolation on anxiety-like behaviors, depressive-like behaviors, social interaction, and cognitive function by novelty suppressed feeding test, elevated plus maze test, open field test, social interaction test, sucrose preference test, forced swim test, tail suspension test, and novel object recognition test, respectively (Figure 1A). Behavioral tests showed that female mice exposed to social isolation, the latency to feeding in NSFT was significantly increased (Figure 1B, *p* = 0.0005, Group median: 99.00; Isolation median: 417.5, Mann-Whitney test). The total traveled distance in OFT (an index of locomotor) was not changed (Figure 1C, *t*_(19)_ *=* 0.952, *p* = 0.35) but time in the center area was decreased (Figures 1D,E, *t*_(19)_ *=* 2.822, *p* = 0.01). In the EPM test, time in open arms was decreased (Figure 1F, *t*_(22)_ *=* 3.147, *p* = 0.005) with no changes of open arms entries (Figure 1G, *t*_(22)_ *=* 2.039, *p* = 0.05). These data revealed that social isolation induced anxiety-like behaviors in female mice. Additionally, social isolation decreased the SI index of female mice in the social interaction test (Figures 1H,I, *t*_(18)_ *=* 3.386, *p* = 0.003), indicating that isolation induced social anxiety in female mice. Moreover, sucrose preference (Figure 1J, *t*_(22)_ *=* 0.655, *p* = 0.519), immobility in FST (Figure 1K, *t*_(22)_ *=* 0.677, *p* = 0.51), and immobility in TST (Figure 1L, *t*_(22)_ *=* 1.107, *p* = 0.280) were not changed, suggesting that social isolation did not affect depressive-like behaviors in female mice. Novel object recognition index in isolated female mice was not changed compared with group rearing mice (Figure 1M, *t*_(10)_ *=* 0.263, *p* = 0.79), which showed that social isolation did not alter the cognitive performance of female mice. In order to determine the sex differences in mental disorders induced by social isolation, we used a balanced design for male mice. Behavioral tests of male mice showed that the latency to feeding (Figure 1N, *t*_(10)_ *=* 0.951, *p* = 0.36), the total traveled distance in OFT (Figure 1O, *t*_(14)_ *=* 0.530, *p* = 0.60), the time spent in the center area (Figures 1P,Q, *t*_(14)_ *=* 1.171, *p* = 0.16), time in open arms (Figure 1R, *t*_(10)_ *=* 1.494, *p* = 0.16) and open arms entries (Figure 1S, *t*_(10)_ *=* 0.639, *p* = 0.54) in EPM were not affected by social isolation, indicating that chronic social isolation did not induce anxiety-like behaviors in male mice. In addition, social isolation did not change the SI index (Figures 1T,U, *p* = 0.94, Group median:1.915; Isolation median: 1.756, Mann-Whitney test), sucrose preference (Figure 1V, *t*_(10)_ *=* 1.679, *p* = 0.12), immobility in FST (Figure 1W, *t*_(10)_ *=* 1.832, *p* = 0.09), and immobility in TST (Figure 1X, *t*_(10)_ *=* 0.179, *p* = 0.86), showing that social isolation had no effects on social anxiety and depressive-like behaviors in male mice. Notably, isolated male mice showed an increased recognition index measured in NORT (Figure 1Y). These data confirmed that female mice were more susceptible to childhood social isolation induced anxiety-like behaviors.

### Childhood social isolation elevated peripheral inflammation in female mice

**FIGURE 2:**
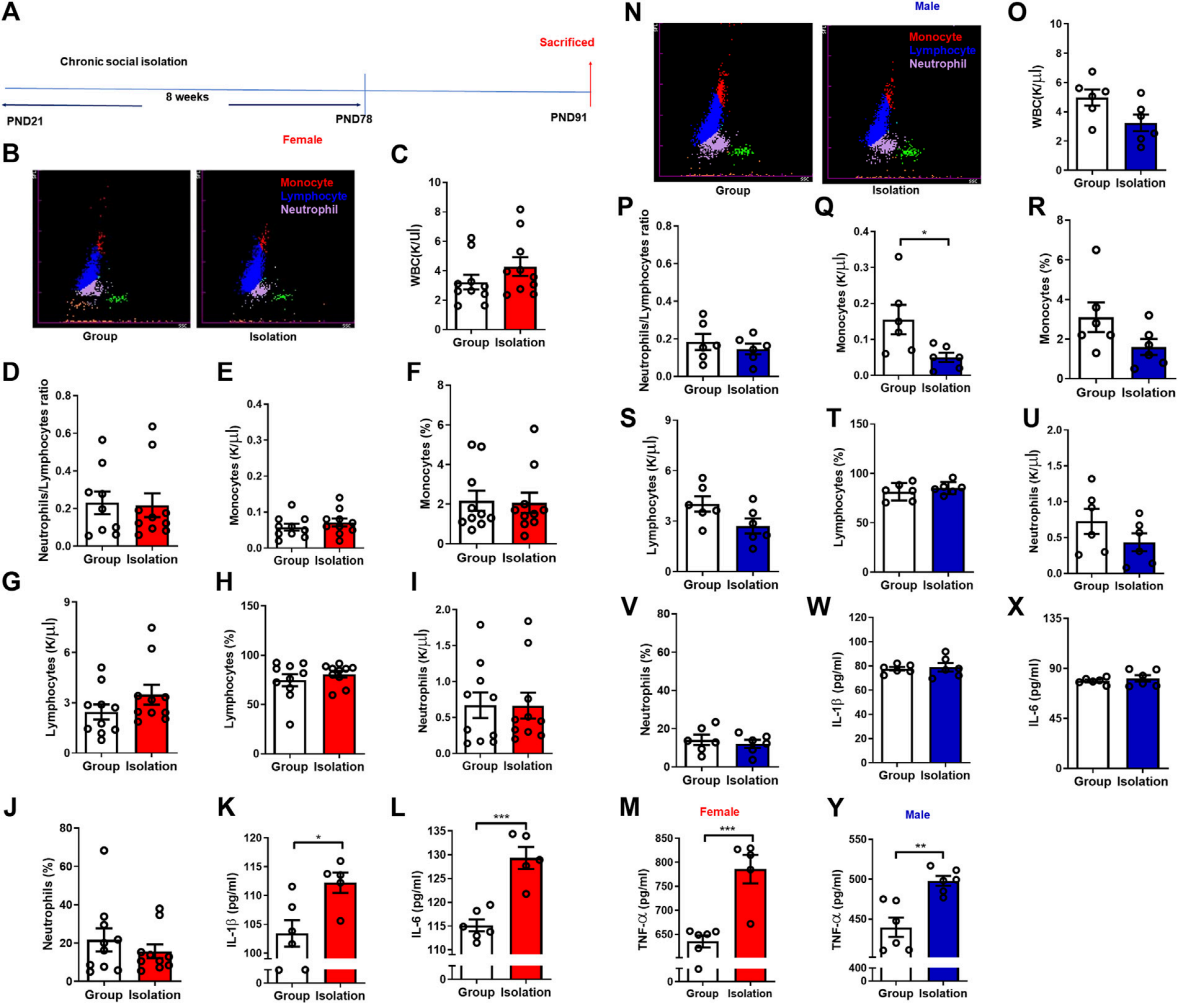
Childhood social isolation elevated peripheral inflammation in female mice. **(A)** Timeline of the experimental procedure. **(B)** Representative images of five-classification blood cell counts of female mice, and various WBC groups were represented by different colors: monocytes (red), lymphocytes (blue) and neutrophils (purple). **(C)** The total counts of WBC in female mice (red bars, *n* = 10). **(D)** The neutrophil/lymphocyte ratio. **(E)** The monocytes counts. **(F)** The monocytes percentage. **(G)** The lymphocytes counts. **(H)** The lymphocyte percentage. **(I)** The neutrophils counts. **(J)** The neutrophils percentage. **(K)** The levels of IL-1β, **(L)** IL-6, **(M)** TNF-α. **(N)** Representative images of five classification blood cell counts of male mice. **(O)** The total counts of WBC in male mice (blue bars, *n* = 6). **(P)** The neutrophil/lymphocyte ratio. **(Q)** The monocytes counts. **(R)** The monocytes percentage. **(S)** The lymphocytes counts. **(T)** The lymphocytes percentage. **(U)** The neutrophils counts. **(V)** The neutrophils percentage. **(W)** The levels of IL-1β, **(X)** IL-6, **(Y)** TNF-α. Data were mean ± SEM, *p < 0.05, **p < 0.01, ***p < 0.001.

Peripheral inflammation levels are elevated after social isolation, mediating anxiety- and depressive-like behaviors (Krügel et al., 2014). To investigate whether inflammation is responsible for increased vulnerability to social isolation, we next measured the levels of peripheral inflammatory markers after chronic social isolation in both males and females (Figure 2A). Chronic childhood social isolation did not change the levels of various types of WBCs in female mice as indicated by unchanged WBCs total counts (Figures 2B,C, *t*_(18)_ *=* 1.312, *p* = 0.21), neutrophils/lymphocytes ratio (Figure 2D, *t*_(17)_ *=* 0.985, *p* = 0.34), number of monocytes (Figure 2E, *t*_(18)_ *=* 0.908, *p* = 0.38), percentage of monocytes (Figure 2F, *t*_(18)_ *=* 0.153, *p* = 0.88), number of lymphocytes (Figure 2G, *t*_(18)_ *=* 1.380, *p* = 0.18), percentage of lymphocytes (Figure 2H, *t*_(18)_ *=* 0.871, *p* = 0.39), number of neutrophils (Figure 2I, *t*_(18)_ *=* 0.238, *p* = 0.98), and the percentage of neutrophils (Figure 2J, *t*_(18)_ *=* 0.845, *p* = 0.41). It has been verified by *t*-test that isolation did not affect the WBCs of female mice. We next examined the pro-inflammatory cytokines and found that the levels of plasma IL-1β (Figure 2K, *t*_(9)_ *=* 2.944, *p* = 0.02), IL-6 (Figure 2L, *t*_(9)_ *=* 5.654, *p* = 0.0003) and TNF-α (Figure 2M, *t*_(9)_ *=* 5.010, *p* = 0.0007) were significantly higher in female mice undergoing isolation than group rearing mice. These data indicate that activated peripheral inflammation is possibly linked to female sensitivity to social isolation.

We also performed WBCs analysis and pro-inflammatory cytokines assay in male mice to uncover the potential sex difference of social isolation on peripheral inflammation. We found that after social isolation, the number of WBCs (Figures 2N,O, *t*_(10)_ *=* 2.184, *p* = 0.05) and neutrophils/lymphocytes ratio (Figure 2P, *t*_(10)_ *=* 0.729, *p* = 0.48) were not significantly changed in male mice. While the number of monocytes (Figure 2Q, *t*_(10)_ *=* 2.449, *p* = 0.03) was decreased significantly. Moreover, the percentage of monocytes (Figure 2R, *t*_(10)_ *=* 1.764, *p* = 0.11), number of lymphocytes (Figure 2S, *t*_(10)_ *=* 2.066, *p* = 0.07), percentage of lymphocytes (Figure 2T, *t*_(10)_ *=* 0.846, *p* = 0.42), number of neutrophils (Figure 2U, *t*_(10)_ *=* 1.362, *p* = 0.20) and the percentage of neutrophils (Figure 2V, *t*_(10)_ *=* 0.607, *p* = 0.56) were not significantly changed. The levels of IL-1β (Figure 2W, *t*_(10)_ *=* 0.356, *p* = 0.73), and IL-6 (Figure 2X, *t*_(10)_ *=* 0.739, *p* = 0.48) were not changed in isolated male mice, while the level of TNF-α (Figure 2Y, *t*_(10)_ *=* 4.264, *p* = 0.002) was higher in isolation than group rearing mice.

### Childhood social isolation elevated neuroinflammation in amygdala in female mice

**FIGURE 3:**
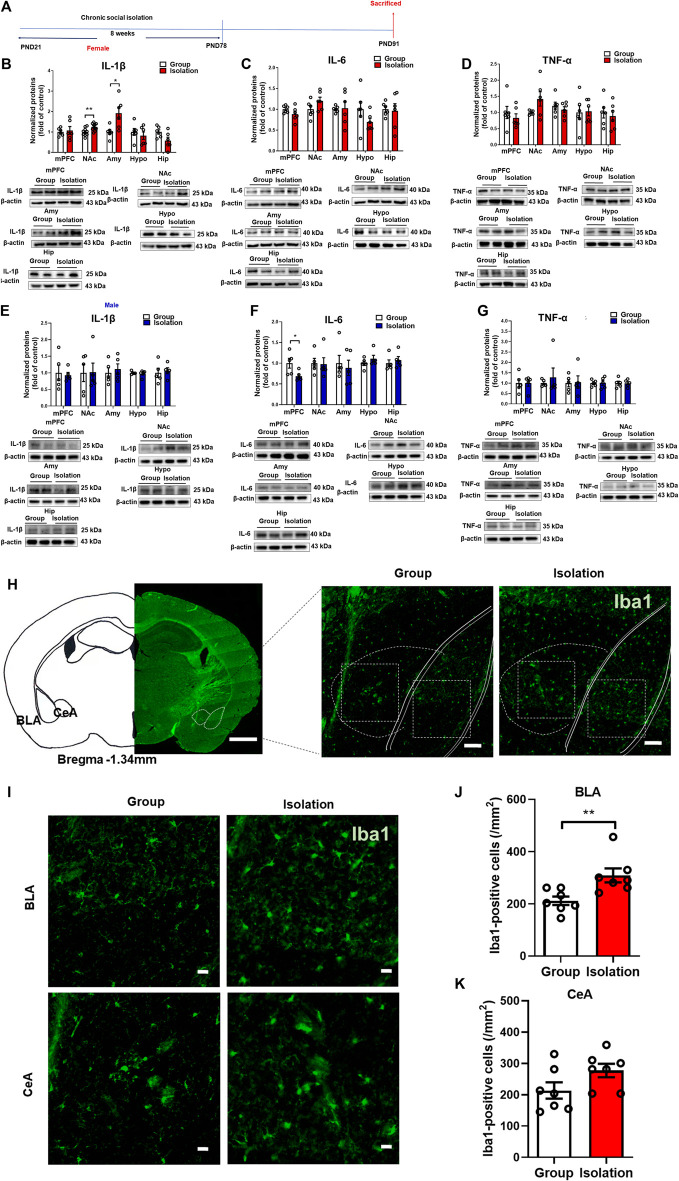
Childhood social isolation elevated neuroinflammation in amygdala in female mice. **(A)** Timeline of the experimental procedure. **(B)** The expressions of IL-1β, **(C)** IL-6, **(D)** TNF-α in the mPFC, NAc, Amy, Hypo and Hip of female mice (red bar, *n* = 6) and representative immunoblots shown on the bottom of bar graphs. **(E)** The expressions of IL-1β, **(F)** IL-6, **(G)** TNF-α in the mPFC, NAc, Amy, Hypo and Hip of male mice (blue bar, *n* = 5) with representative immunoblots on the bottom of bar graphs. **(H)** Representative images of amygdala and subareas (BLA and CeA). Scale bar = 1 mm (location diagram of amygdala) and 100 μm (partial schematic diagram). **(I)** Representative images of Iba-1 (a microglia marker) staining in amygdala, scale bar = 20 μm (bottom). **(J,K)** Social isolation increased microglia in BLA but not in CeA, *n* = 3. Data were mean ± SEM, *p < 0.05, **p < 0.01. mPFC, medial prefrontal cortex; NAc, nucleus accumbens; Amy, amygdala; Hypo, hypothalamus; Hip, hippocampus.

The above results showed that the development of anxiety-like behaviors was influenced by the activation of peripheral inflammation during chronic social isolation in female mice. To further examine how neuroinflammation affects the susceptible response to social isolation in female mice, we detected the pro-inflammatory cytokines IL-1β, IL-6, and TNF-α levels in mPFC, NAc, Amy, Hypo and Hip (Figure 3A). In female mice, Western blot assay showed that compared with group mice, the mPFC, Hypo and Hip (Figure 3B; *t*_(10)_ *=* 0.731, *p* = 0.48; *t*_(10)_ *=* 2.134, *p* = 0.06; *t*_(10)_ *=* 0.366, *p* = 0.72, respectively) IL-1β expression levels were unchanged in social isolation. However, in the NAc and Amy, IL-1β expression levels were increased (Figure 3B; *t*_(18)_ = 3.058, *p* = 0.007; *t*_(10)_ *=* 2.862, *p* = 0.02, respectively). The levels of IL-1β were increased by 91.4% in Amy after isolation and increased by 24.9% in NAc. The expressions of IL-6 in the mPFC, NAc, Amy, Hypo and Hip were not significantly changed after isolation (Figure 3C; *t*_(10)_ *=* 1.489, *p* = 0.17; *t*_(10)_ *=* 1.676, *p* = 0.12; *t*_(10)_ *=* 0.148, *p* = 0.89; *t*_(10)_ *=* 1.486, *p* = 0.17; *t*_(10)_ *=* 0.203, *p* = 0.84, respectively). TNF-α expressions in these regions were not significantly changed after social isolation (Figure 3D; *t*_(10)_ *=* 0.737, *p* = 0.48; *p* = 0.31, Group median: 1.000; Isolation median: 2.042, Mann-Whitney test; *t*_(10)_ *=* 0.781, *p* = 0.45; *t*_(10)_ *=* 0.135, *p* = 0.89; *t*_(10)_ *=* 0.45, *p* = 0.66, respectively).

In males, IL-1β expression levels in the mPFC, NAc, Amy, Hypo and Hip (Figure 3E; *t*_(8)_ *=* 0.291, *p* = 0.78; *t*_(8)_ *=* 0.054, *p* = 0.96; *t*_(8)_ *=* 0.509, *p* = 0.62; *t*_(8)_ *=* 0.408, *p* = 0.69; *t*_(8)_ *=* 0.376, *p* = 0.72, respectively) were unchanged in isolated mice. The IL-6 expressions in the NAc, Amy, Hypo and Hip were also not significantly changed after isolation (Figure 3F, *t*_(8)_ *=* 0.122, *p* = 0.91; *t*_(8)_ *=* 0.420, *p* = 0.69; *t*_(8)_ *=* 1.120, *p* = 0.29; *t*_(8)_ *=* 0.119, *p* = 0.91, respectively), whereas IL-6 expression in the mPFC was decreased after isolation (Figure 3F, *t*_(8)_ *=* 2.525, *p* = 0.04). TNF-α expressions in the mPFC, NAc, Amy, Hypo and Hip were not significantly changed after isolation (Figure 3G, *t*_(8)_ *=* 0.044, *p* = 0.97; *t*_(8)_ *=* 0.6217, *p* = 0.55; *t*_(8)_ *=* 0.149, *p* = 0.89; *t*_(8)_ *=* 0.119, *p* = 0.91; *t*_(8)_ *=* 0.048, *p* = 0.96, respectively). These data suggested that socially isolated female mice exhibited increased levels of neuroinflammation in the amygdala compared with the group rearing mice, as manifested by significantly elevated levels of the pro-inflammatory cytokine IL-1β. Therefore, we further detected microglia in the amygdala by immunofluorescence staining (Figure 3H). The results showed that the number of Iba-1-positive cells in the basolateral amygdala (BLA) was significantly increased (Figures 3I,J, *t*_(12)_ *=* 3.112, *p* = 0.009), although the difference of Iba-1-positive cells in the central amygdala (CeA) was not statistically significant (Figure 3K, *t*_(12)_ *=* 1.890, *p* = 0.08), indicating that the microglia in the BLA was activated. Taken together, social isolation induces BLA-specific neuroinflammation as manifested by markedly elevated levels of IL-1β and increased numbers of microglia in female mice.

### Childhood social isolation damaged BBB in amygdala in female mice

**FIGURE 4:**
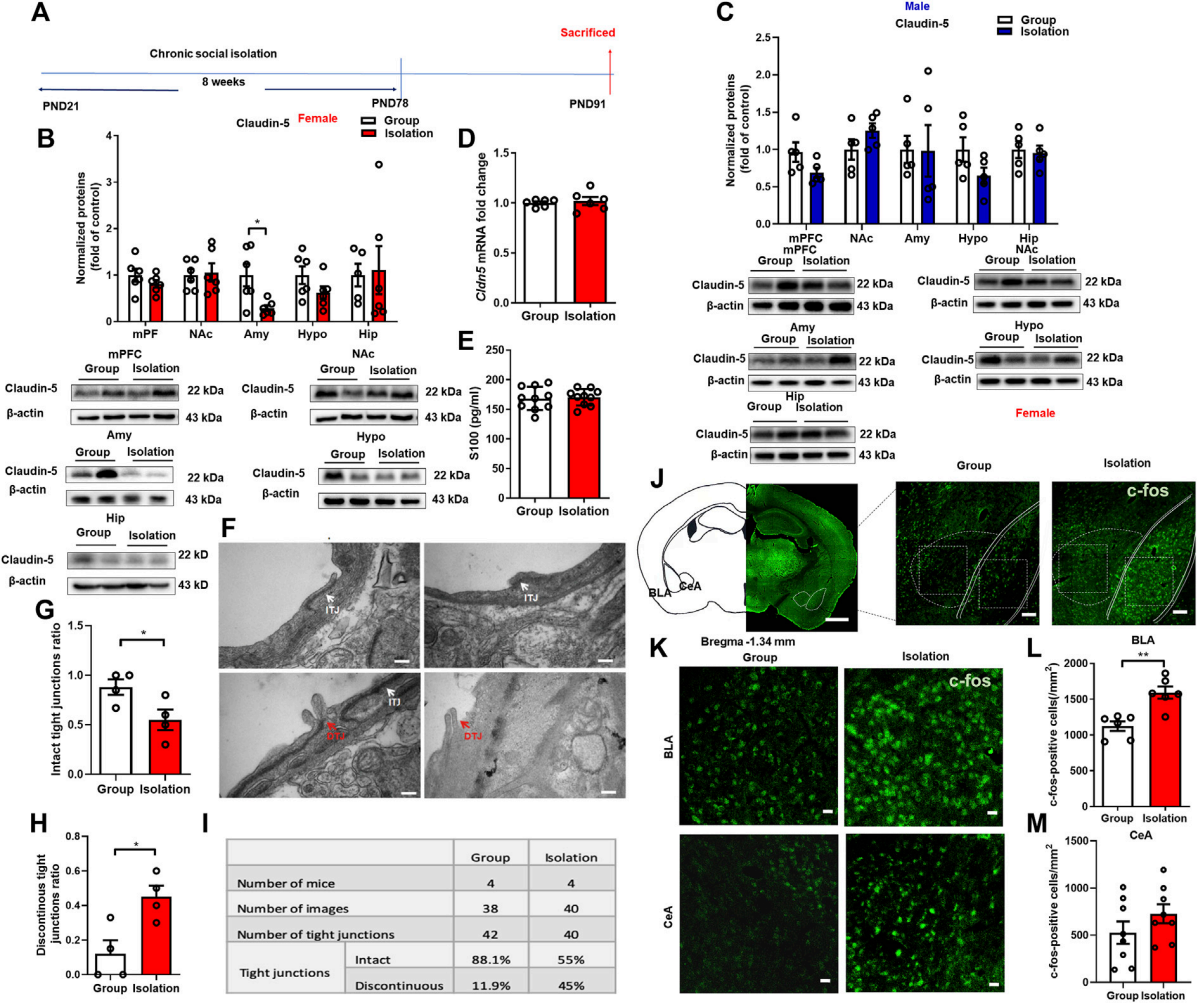
Childhood social isolation damaged BBB in amygdala in female mice. **(A)** Timeline of the experimental procedure. **(B)** The levels of Claudin-5 in the mPFC, NAc, Amy, Hypo and Hip of female mice (red bars, *n* = 6) and representative immunoblots. **(C)** The levels of Claudin-5 in the mPFC, NAc, Amy, Hypo and Hip of male mice (blue bars, *n* = 5) and representative immunoblots. **(D)** Quantitative PCR measurement of *Cldn5* mRNA. **(E)** The level of S100 measured by ELISA. **(F)** The intact tight junctions (white arrows) and discontinuous tight junctions (red arrows) measured by transmission electron microscopy. Scale bar = 200 nm. **(G)** The intact tight junctions ratio **(H)** The discontinuous tight junctions ratio. **(I)** Summary of tight junctions in the amygdala of female mice. **(J)** Representative images of c-fos in the amygdala (BLA and CeA). Scale bar = 1 mm (location diagram of amygdala) and 100 μm (partial schematic diagram). **(K)** Representative images of c-fos immunofluorescence staining in BLA and CeA, scale bar = 20 μm. **(L,M)** Quantitative assay of c-fos positive cells in BLA and CeA, *n* = 3. Data were mean ± SEM, *p < 0.05, **p < 0.01. mPFC, medial prefrontal cortex; NAc, nucleus accumbens; Amy, amygdala; Hypo, hypothalamus; Hip, hippocampus.

The BBB is a bridge between peripheral inflammation and neuroinflammation. Stress-induced BBB damage has been confirmed to be associated with anxiety- and depressive-like behaviors with differential characteristics in males and females (Esposito et al., 2001; Liu et al., 2012; Wohleb et al., 2013). To better understand whether the deficits of BBB contribute to the increased neuroinflammation in the amygdala and the vulnerability to chronic social isolation in female mice, we next assessed the tight junction protein Claudin-5, a gatekeeper of BBB permeability, in the above five brain regions (Figure 4A). Female mice received social isolation, the Claudin-5 expressions in the mPFC, NAc, Hypo and Hip (Figure 4B, *t*_(10)_ *=* 1.156, *p* = 0.27; *t*_(10)_ *=* 0.256, *p* = 0.80; *t*_(10)_ *=* 1.623, *p* = 0.14; *t*_(9)_ *=* 0.172, *p* = 0.87) were not significantly changed. In contrast, the Claudin-5 in Amy was significantly decreased (Figure 4B, *t*_(9)_ *=* 3.384, *p* = 0.008). In male mice, social isolation did not affect Claudin-5 expression in the mPFC, NAc, Amy, Hypo and Hip (Figure 4C, *t*_(8)_ *=* 1.498, *p* = 0.09, *t*_(8)_ *=* 1.498, *p* = 0.17; *t*_(8)_ *=* 0.048, *p* = 0.96; *t*_(8)_ *=* 1.799, *p* = 0.11; *t*_(8)_ *=* 0.319, *p* = 0.76). The mRNA expression level of *Cldn5* in Amy was detected by qPCR, and the data showed that there was no significant change in the mRNA levels of *Cldn5* mRNA in female isolated mice compared with the group rearing group (Figure 4D, *t*_(10)_ *=* 0.448, *p* = 0.66). We also used ELISA to detect the BBB damage marker S100 in the plasma of female mice, and the results showed that there was no significant difference between the isolation and the group rearing mice (Figure 4E, *t*_(18)_ *=* 0.28, *p* = 0.78). The results from transmission electron microscope for assessment of the ultrastructure of tight junctions in amygdala showed that after social isolation, the proportion of discontinuous tight junction (DTJ) was increased significantly (Figures 4F,G, *t*_(6)_ = 3.249, *p* = 0.02), while the level of intact tight junction (ITJ) decreased significantly (Figures 4F,H, *t*_(6)_ = 2.532, *p* = 0.05). In group housed female mice, intact tight junctions accounted for 88.1% of all observed tight junctions, and discontinuous tight junctions accounted for 11.9%. But in isolated female mice, intact tight junctions accounted for 55% of all observed tight junctions, and discontinuous tight junctions accounted for 45% (Figure 4I). These results suggest that the tight junction integrity of BBB in the amygdala is disrupted by social isolation in female mice.

Neuronal activity is regulated by the inflammatory cytokine IL-1β and microglia, which in turn regulates microglia activation, consequently affecting BBB permeability (Bradford, 1995; Vazana et al., 2016). Therefore, we used immunofluorescence staining of c-fos to detect the changes of amygdala neuronal activity in female mice. The results showed that c-fos expression in the BLA of female mice was significantly increased (Figures 4J–L, *t*_(10)_ *=* 4.361, *p* = 0.001), confirming increased neuronal activity in this brain region. However, there was no significant difference in c-fos expression in the CeA (Figures 4J–M *t*_(14)_ *=* 1.289, *p* = 0.22).

### Down-regulation of Claudin-5 in amygdala induced anxiety-like behavior in female mice

**FIGURE 5:**
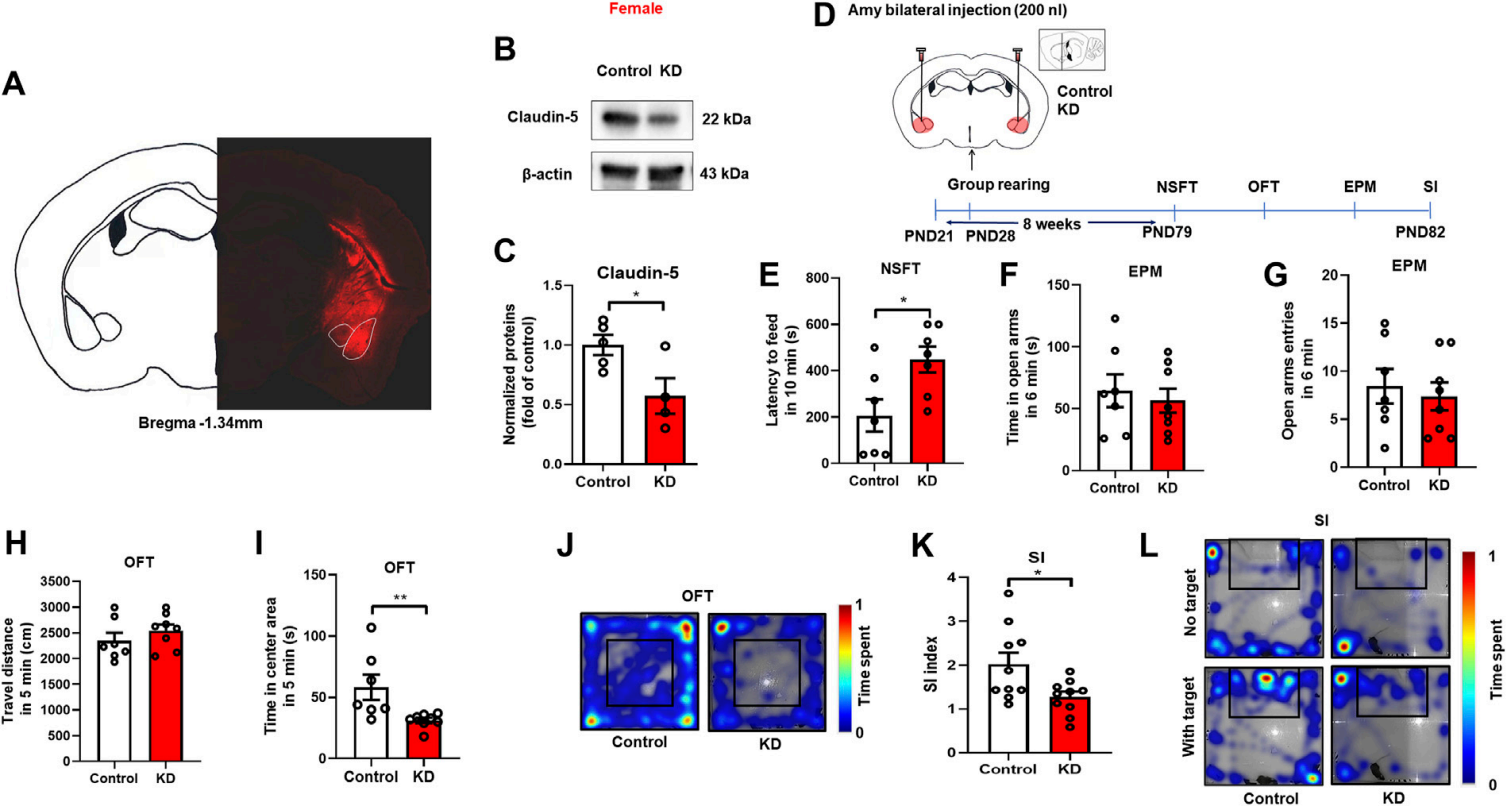
Down-regulation of Claudin-5 expression in the amygdala produced anxiety-like behavior in female mice. **(A)** Representative images of AAV-infected cells in the amygdala of female mouse brain coronal sections (-1.34 mm relative to bregma). Scale bar = 1 mm. **(B)** Immunoblots of AAV9-CAG-shRNA-mCherry (Control) or AAV-CAG-*Cldn5*-shRNA-mCherry (KD) in the amygdala. **(C)** Quantitative assay of Claudin-5 (*n* = 4–5). **(D)** Experimental timeline. **(E)** The novelty suppressed feeding test. **(F,G)** The time and entries in open arms in the elevated plus maze test. **(H)** The locomotor activity in the open filed test. **(I)** The time in the center area of open field test. **(J)** The representative heat maps during the open field test. **(K)** The social interaction test. **(L)** The representative heat maps of social interaction test. (*n* = 7–10). Data were mean ± SEM, *p < 0.05, **p < 0.01.

Given the findings that decreased expression of Claudin-5 in the amygdala is linked to isolation-induced anxiety-like behaviors in females, we further investigated whether down-regulation of Claudin-5 in the amygdala could induce anxiety-like behaviors in group-housed female mice. Firstly, the protein knockdown effect of the adeno-associated virus (AAV) short hairpin RNA (shRNA) was verified. Stereotactic injection of knockdown (KD) virus AAV-CAG-*Cldn5*-shRNA-mCherry or negative control (Control) virus AAV9-CAG-shRNA-mCherry in the amygdala (Figure 5A). After 4 weeks of expression, Western blot results showed that the KD virus reduced the expression of Claudin-5 in the amygdala (Figures 5B,C, *t*_(7)_ *=* 2.649, *p* = 0.03). In the experiment on down-regulation of Claudin-5, female mice were group rearing from PND 21, and stereotaxic surgery was performed at 4 weeks of age. The mice were divided into two groups, the Control group, and the KD group, and were injected with the negative control virus AAV9-CAG-shRNA-mCherry or adeno-associated shRNA interference virus AAV-CAG-*Cldn5*-shRNA-mCherry into the amygdala, respectively. On PND 79, behavioral tests were performed (Figure 5D). The results showed that down-regulation of Claudin-5 in the amygdala significantly increased the latency to feeding in NSFT (Figure 5E, *t*_(12)_ *=* 2.72, *p* = 0.02). In the EPM test, down-regulation of Claudin-5 did not affect the time in the open arms (Figure 5F, *t*_(13)_ *=* 0.49, *p* = 0.63) and entries into the open arm (Figure 5G, *t*_(13)_ *=* 0.459, *p* = 0.65). In OFT, down-regulation of Claudin-5 did not alter the locomotor in female mice (Figure 5H, *t*_(13)_ *=* 0.994, *p* = 0.34) but caused a decrease of time in the center area (Figures 5I,J, *p* = 0.004, Group median: 44.00; Isolation median: 32.50, Mann-Whitney test). The above results indicated that down-regulation of Claudin-5 expression in the amygdala induced anxiety-like behaviors in female mice. In the social interaction test, down-regulation of Claudin-5 caused a significant decrease in the SI index of female mice (Figures 5K,L, *t*_(18)_ *=* 2.539, *p* = 0.02), indicating that female mice exhibited social anxiety behavior.

### Overexpression of Claudin-5 in amygdala prevented anxiety-like behaviors induced by social isolation in female mice

**FIGURE 6:**
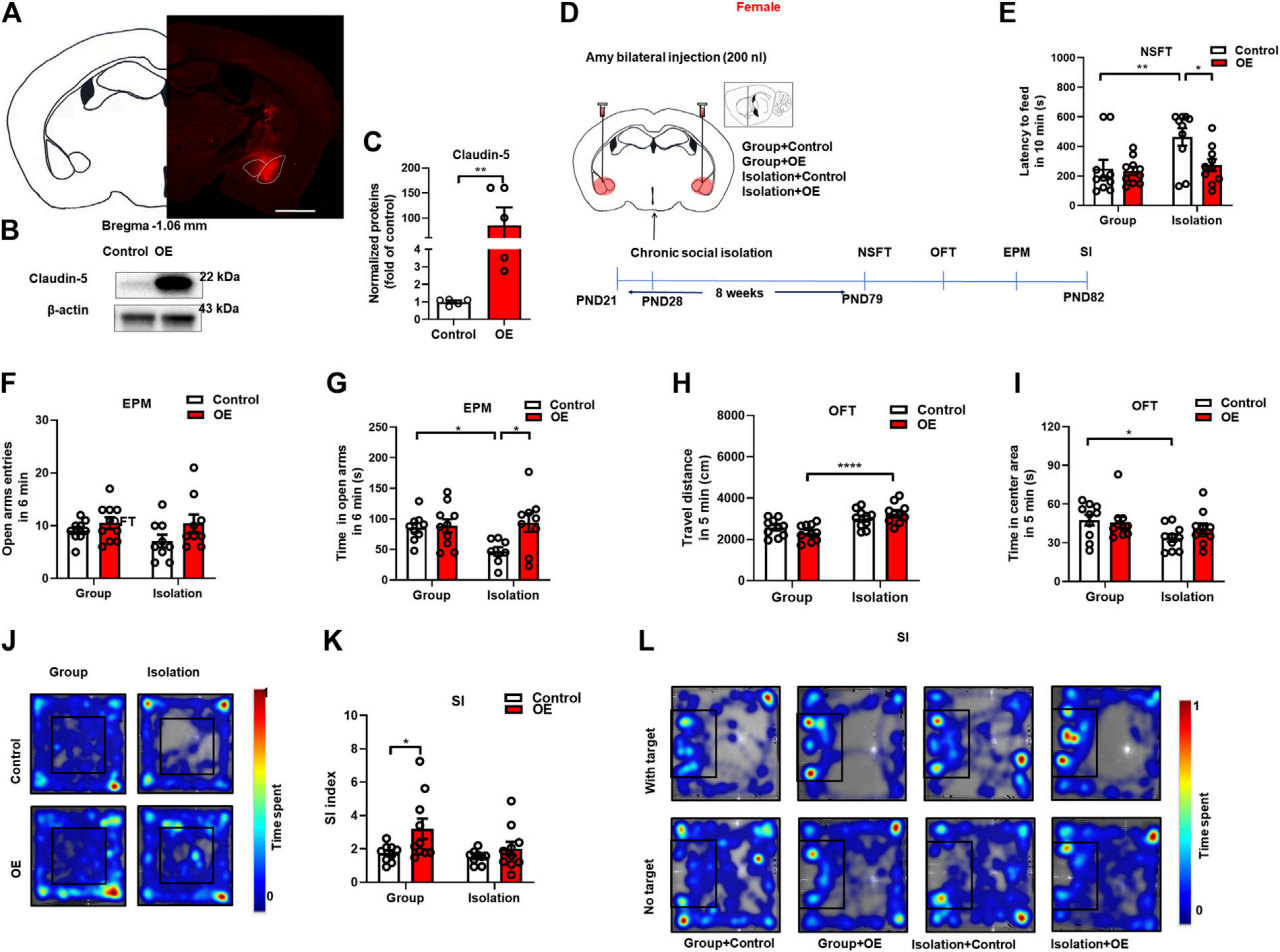
Overexpression of Claudin-5 in the amygdala prevented anxiety-like behavior induced by social isolation in female mice. **(A)** Representative images of AAV-infected cells in the amygdala of female mouse brain coronal sections (−1.06 mm relative to bregma). Scale bar = 1 mm. **(B)** Immunoblot analyses of AAV9-CAG-mCherry (Control) or AAV9-CAG-*Cldn5*-mCherry (OE) expression in the amygdala. **(C)** Quantitative assay of Claudin-5 (*n* = 5). **(D)** Experimental procedure. **(E)** The novelty suppressed feeding test. **(F,G)** The time and entries in open arms in the elevated plus maze test. **(H)** The locomotor activity in the open filed test. **(I,J)** The time in the center area and representative heat maps of open field test. **(K,L)** The social interaction test and representative heat maps. (*n* = 8–10), Two-way ANOVA, Bonferroni’s test. Data were mean ± SEM, *p < 0.05, **p < 0.01, ****p < 0.0001.

From the above results, we found that down-regulation of Claudin-5 increased anxiety-like behaviors in group-housed female mice, suggesting that amygdala BBB damage may be a key factor in isolation-induced anxiety-like consequences in female mice. Here, to further determine the role of BBB tight junctions in anxiety, we investigated whether up-regulation of Claudin-5 expression in the amygdala could prevent isolation-induced anxiety-like behaviors in females. First, we assessed the effects of the constructed AAV overexpression vector by injecting the overexpression (OE) virus AAV-CAG-*Cldn5*-mCherry or the negative control AAV-CAG-mCherry (Control) into BLA (Figure 6A). After 4 weeks of stable virus expression, Western blot assay showed that AAV overexpression could significantly increase Claudin-5 in the amygdala (*p* = 0.0079, Group median: 1.100; Isolation median: 100.5, Mann-Whitney test) (Figures 6B,C). In the experiment of Claudin-5 overexpression, female mice were housed in group or in isolation from 3 weeks of age, and stereotaxic surgery was performed at 4 weeks of age. Then group rearing and isolation mice were divided into two groups, injected by control or OE virus respectively, namely Group + Control, Group + OE, Isolation + Control, and Isolation + OE. The adeno-associated overexpression virus AAV-CAG-*Cldn5*-mCherry or the negative control virus AAV-CAG-mCherry were bilaterally injected into BLA, respectively, and behavioral tests were started at 7 weeks after the operation (Figure 6D). Results indicated that overexpression of Claudin-5 in the amygdala could prevent the social isolation-induced increase of latency to feeding in NSFT. The statistical analysis revealed significant effects of isolation (*F*_(1,36)_ = 6.877, *p* = 0.01) and Claudin-5 (*F*_(1,36)_ = 4.615, *p* = 0.04), but no significant effect of isolation × Claudin-5 interaction (*F*_(1,36)_ = 4.024, *p* = 0.08) for the latency to feeding in NSFT. Comparing with Group + Control, the latency to feeding of the Isolation + Control group was significantly increased (*p* = 0.007) and relative to Isolation + Control group, Isolation + OE group had significantly decreased latency to feeding in novel environment (*p* = 0.02) (Figure 6E). EPM test data revealed significant effects of Claudin-5 (*F*_(1,32)_ = 5.259, *p* = 0.03) but no effects of isolation (*F*_(1,32)_ = 2.360, *p* = 0.13) and isolation × Claudin-5 interaction (*F*_(1,32)_ = 4.094, *p* = 0.05) for the time in the open arms. Compared with Group + Control, the time in open arms of the Isolation + Control group was significantly decreased (*p* = 0.04), and the time in open arms was significantly increased in the Isolation + OE group compared with Isolation + Control (*p* = 0.01) (Figure 6F). There were no significant effects of isolation (*F*_(1,33)_ = 0.379, *p* = 0.06), Claudin-5 (*F*_(1,32)_ = 5.259, *p* = 0.03), and isolation × Claudin-5 interaction (*F*_(1,33)_ = 0.717, *p* = 0.43) for the open arms entries (Figure 6G). These data showed that overexpression of Claudin-5 in the amygdala could prevent the social isolation-induced decrease in the time in the open arms of the EPM. There was a significant effect of isolation (*F*_(1,36)_ = 24.10, *p* < 0.0001), no significant effects of Claudin-5 (*F*_(1,36)_ = 0.000, *p* = 0.98) and isolation × Claudin-5 interaction (*F*_(1,36)_ = 3.249, *p* = 0.08) for the locomotor. The locomotor of the Isolation + OE group was significantly increased compared to the Group + OE group (*p* < 0.0001) (Figure 6H). There was a significant effect of isolation (*F*_(1,36)_ = 5.487, *p* = 0.02), no significant effects of Claudin-5 (*F*_(1,36)_ = 0.475, *p* = 0.49) and isolation × Claudin-5 interaction (*F*_(1,36)_ = 1.299, *p* = 0.49) for the time in the center area in the open field test. Compared with the Group + Control group, time in the central area of the open field in the Isolation + Control group was significantly decreased (*p* = 0.04), but compared with the Group + OE group, there was no significant difference in the time spent in the central area of the open field in the Isolation + OE group (*p* = 0.80), indicating that the overexpression of Claudin-5 in the amygdala increased time in the central area (Figures 6I,J). In the social interaction test, there were significant effects of Claudin-5 (*F*_(1,34)_ = 5.863, *p* = 0.02) but no significant effects of isolation (*F*_(1,34)_ = 3.032, *p* = 0.09) and isolation × Claudin-5 interaction (*F*_(1,34)_ = 1.405, *p* = 0.24) for the SI index. Compared with Group + Control, the SI index of Group + OE group increased significantly (*p* = 0.03) (Figures 6K,L), suggesting that Claudin-5 overexpression significantly increased social interaction. Altogether, these data suggest that enhancement of Claudin-5 in the amygdala could prevent isolation-induced anxiety-like behaviors and may serve as a potential therapeutic target for emotional deficits, particularly in female mice.

## Discussion

**FIGURE 7:**
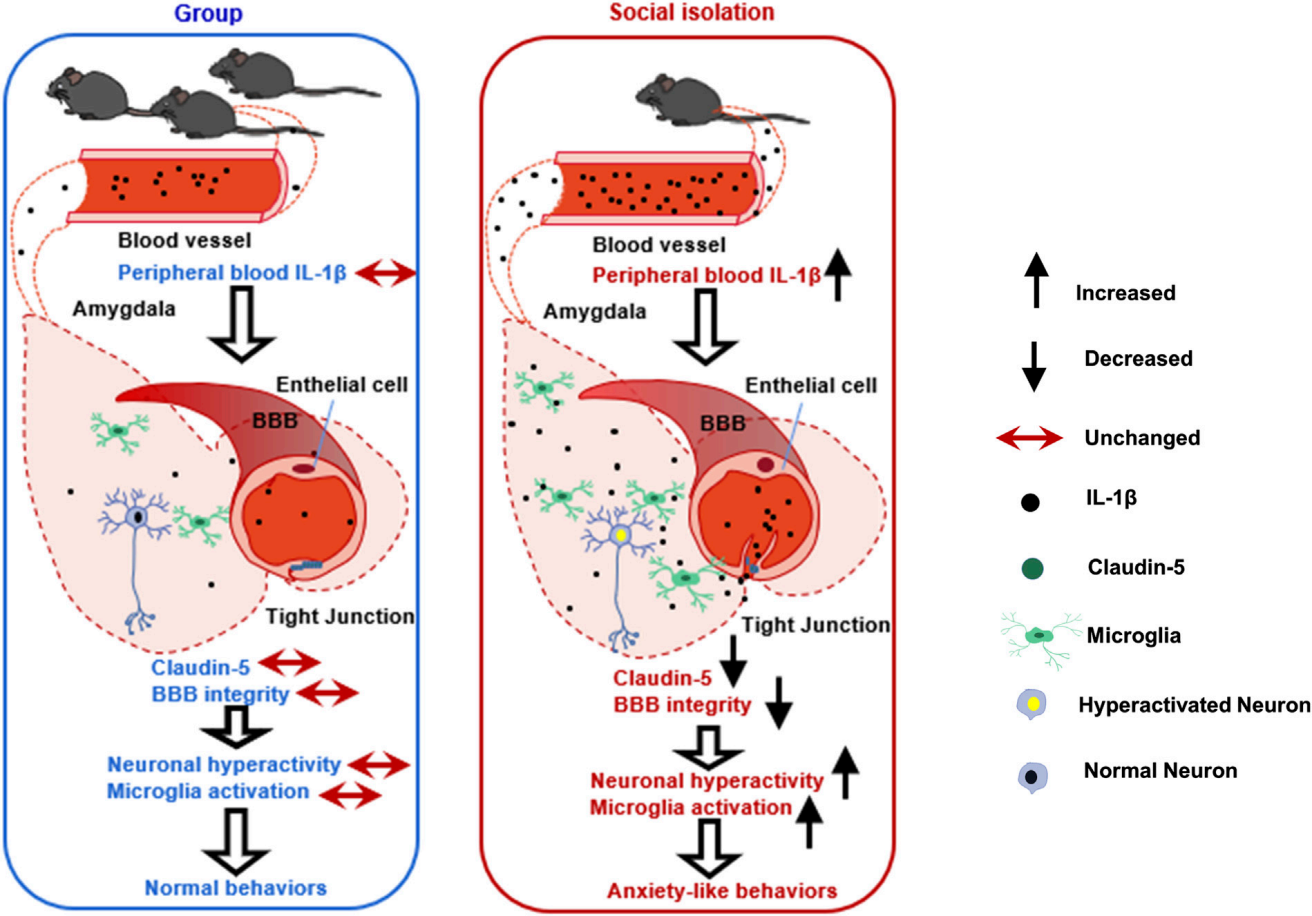
A schematic diagram outlining the mechanisms of impaired BBB in social isolation-induced anxiety-like behaviors in female mice. The left picture shows the normal BBB integrity in the amygdala in group-housed female mice without anxiety-like behaviors. The right picture shows the damaged amygdala BBB in female mice with anxiety-like behaviors after social isolation. We speculate that the possible mechanisms by which female mice experienced social isolation are more susceptible to anxiety is that social isolation-induced peripheral cytokine IL-1β infiltrating into the brain parenchyma, further promoting local BBB leakage, increasing neuroinflammation, and triggering neuronal activation in the amygdala.

In this study, we have demonstrated that after 8 weeks of post-weaning social isolation, female mice show anxiety-like behaviors, while male mice do not demonstrate behavioral alterations. We have also found that the vulnerability to social isolation in female mice is related to the increased peripheral cytokines, neuroinflammation and impaired BBB integrity in the amygdala. In female mice, viral-mediated downregulation of Claudin-5 is sufficient to induce anxiety-like behaviors, while overexpression of Claudin-5 prevents these behavioral deficits induced by social isolation (Figure 7).

Social isolation, as psychosocial stress, has been found to induce anxiety-, depressive-like behaviors, and changes in social interaction and cognitive performance (Albus, 2010; Liu et al., 2020; Sepúlveda-Loyola et al., 2020; Smith et al., 2020; Cauberghe et al., 2021; Fruehwirth et al., 2021; Morrissette, 2021). Among socialized mammals, females are more prosocial and more prone to abnormal behavioral changes after social isolation (Seltmann et al., 2019; Fukumitsu et al., 2022). Several studies comparing the behavioral effects of social isolation on the two sexes have shown that after experiencing social isolation, female mice are more likely to exhibit anxiety-, depressive-like behaviors, social anxiety, and irreversible cognitive impairment, while male animals are more resistant to social isolation instead of aggressive behaviors (Huang et al., 2017; Hinton et al., 2019; Tan et al., 2021). Consistent with previous studies, we showed that female mice developed anxiety-like behaviors after 8 weeks of social isolation, suggesting that female mice were more sensitive to social isolation regarding the anxiety-like phenotypes. Some previous studies have shown that early social isolation may lead to increased locomotor, and the increased exploratory behavior in unfamiliar environments may explain that social isolation induces mice in a more cautious and defensive state (Lander et al., 2017; Menard et al., 2017; Dion-Albert et al., 2022).

Previous studies revealed that both female and male mice exhibit depressive-like behaviors after experiencing early-life social isolation, while male mice also exhibit anxiety-like behaviors (Kokare et al., 2010; Salihu et al., 2021). In addition, the findings are inconsistent concerning the impact of isolation on mouse social and cognition. Some studies revealed that social isolation causes increased social craving in female and male mice, as well as others showed that social isolation causes social anxiety (Pais et al., 2019; Rivera-Irizarry et al., 2020; Yamamuro et al., 2020). Moreover, some studies suggest that social isolation can impair cognitive function in mice, while others suggest that social isolation improves cognitive performance in mice (Ren et al., 2015; Fei et al., 2019). Our results show that female mice display social anxiety after chronic isolation, while male mice have better cognitive function than controls. The conclusions of previous studies on the abnormal behaviors of mice caused by early social isolation are quite different, showing inconsistency with the results of this study to some extents. This may be due to that there are many factors that affect the results of behavioral tests, including differences in the social isolation model, rearing environments, behavioral testing indicators, and testing methods, which play vital roles in the behavioral performance of mice.

Regarding the mechanism of sex differences in social isolation, previous studies have shown that social isolation induced different neural circuit activity changes in both sexes, different effects on neuroplasticity, the hypothalamo-pituitary-adrenal axis, and inflammatory changes in females (Hermes et al., 2006; Keesom et al., 2018; Hinton et al., 2019; Tan et al., 2021). Consistent with previous findings, our results have confirmed that female mice had significantly increased levels of peripheral pro-inflammatory factors, including IL-1β, IL-6 and TNF-α after social isolation (Boscarino and Chang, 1999; Vidović et al., 2011; Zhou et al., 2014). There was no significant change in inflammatory cells in isolated mice. However, the number of leukocytes and lymphocytes showed an increasing trend, which may be related to the anxiety phenotype of female mice. The reason why this study did not show significant changes in WBCs may be due to the WBCs themselves varying greatly in different individuals. In the case of a small sample size, it is difficult to detect the changes of WBCs affected by social isolation accurately.

Comparing with females, male mice exposed to social isolation had a lower inflammatory response, manifested as only elevated TNF-α but not IL-1β and IL-6. In addition, the percentage of the increase was smaller than that of female mice. After social isolation, male mice showed decreased monocytes, suggesting that males may have lower levels of inflammation. In different studies, the results of monocyte changes in mental disorders were not consistent. Most of the studies showed increased levels of monocytes in patients with anxiety and depression, but it has also been confirmed that monocytes in animal models of depression were decreased (Maes et al., 1992; Zorrilla et al., 2001; Mckim et al., 2018b; Mazza et al., 2018). In this study, the decreased level of monocytes possibly is involved in the regulation of pro-inflammatory and anti-inflammatory of male mice, which promotes the reduction of inflammatory response. We also examined the level of neuroinflammation in brain regions closely related to emotional and social interaction, and found that female mice showed increased IL-1β expression in the NAc and amygdala, and an increase of IL-1β expression was higher in the amygdala. Direct microinjection of IL-1β or TNF-α into several brain regions induces anxiety-, depressive-like behavior, and cognitive dysfunction (Beumer et al., 2012). IL-1β has been confirmed to be a critical factor in neuroinflammation-induced anxiety-like behavior (Mckim et al., 2018a; Mckim et al., 2018b). Stress, a critical trigger to induce anxiety, causes microglia to recruit a large number of monocytes into the brain parenchyma and promotes the synthesis of IL-1 receptor 1 (IL-1R1) in the vascular endothelium (Mckim et al., 2018a; Mckim et al., 2018b). Residential microglias secrete more IL-1β and bind to IL-1R1 in the vascular endothelium to mediate anxiety-like behaviors (Mckim et al., 2018a; Mckim et al., 2018b). Depleting of microglia with minocycline, or knockdown of IL-1R1, could reverse anxiety-like behavior (Mckim et al., 2018a; Mckim et al., 2018b). In this study, microglia immunostaining in the amygdala of female mice showed that microglia in the amygdala was significantly increased after social isolation, mainly in the basolateral amygdala. Individuals with anxiety disorders are often full of fears, evolutionarily conserved survival responses, and persistent fears throughout childhood and adolescence are associated with the high risk of developing anxiety in adulthood (Muris et al., 2000). In addition, accumulating evidence by both imaging and behavioral studies have shown engagement of the amygdala in anxiety disorders and the involvement of amygdala in anxiety-like behaviors in rodents (Delgado et al., 2006). For instance, stimulating the amygdala triggers anxiogenic responses, and inactivating the amygdala reduces anxiety behaviors (Janak and Tye, 2015; Tovote et al., 2015). Our findings that social isolation increased IL-1β and microglia activation in the amygdala confirmed this brain region might play a potential role in driving anxiety behavioral responses of isolated mice. There was no increased neuroinflammation in male mice after social isolation, and the levels of IL-6 in the mPFC were even decreased, consistent with the normal behavioral phenotypes in male mice. Previous studies have demonstrated that reducing neuroinflammation levels in the prefrontal cortex, including reducing IL-6, improved working memory in mice (Hylin et al., 2022). The decreased IL-6 in the mPFC of male mice observed in this study may be related to the better cognitive function of male mice after social isolation.

The findings of current study that social isolation caused a decrease in the expression of Claudin-5 protein in the amygdala of female mice, and the tight junction structure in isolated female mice was also more incomplete than that in the group rearing mice. This was consistent with changes in higher neuroinflammation levels in the amygdala of female mice. Previous evidence have shown that peripheral pro-inflammatory cytokines such as IL-1β, IL-6 and TNF-α can actively transport across BBB, inducing microglia, astrocytes and endothelial cells to release local secondary cytokines and triggered neuroinflammation (Erickson et al., 2012; Shimada and Hasegawa-Ishii, 2017). A recent study have reported significant high levels of IL-1β in restraint-stressed rats both in serum and amygdalar tissues, indicating an inflammatory response occurred with increased BBB permeability (Xu et al., 2019). Moreover, it has been confirmed by fluorescent labeled IL-6 intravenous injection that peripheral IL-6 can enter brain with damaged BBB function and increase the local IL-6 levels (Menard et al., 2017). In this study, increased neuroinflammation was also observed in the amygdala where BBB was damaged, possibly due to the entering of elevated peripheral pro-inflammatory cytokines through the damaged BBB in the amygdala during social isolation. Therefore, we proposed that pro-inflammatory cytokines induced local neuroinflammation, leading to greater sensitivity to social isolation of females.

It is worth noting that the changing trend of Claudin-5 mRNA expression level and the protein expression level was not consistent. The mRNA level remained unchanged but the protein was decreased. It has also been reported that the changing trends of Claudin-5 mRNA and protein levels were inconsistent or even opposite. For example, the mRNA level of Claudin-5 was increased in the prefrontal cortex of schizophrenia patients, while the protein expression was decreased (Nishiura et al., 2017). It can be explained that in this process, the cAMP-PKA pathway was activated and induced the increase of Claudin-5 mRNA expression in a PKA-independent manner, and caused the degradation of Claudin-5 protein in a PKA-dependent manner, during which the homeostasis of Claudin-5 protein levels was mainly in the direction of degradation (Nishiura et al., 2017). This suggested that social isolation decreased Claudin-5 protein in female mice by promoting degradation rather than inhibiting transcription. In male mice, the behavioral manifestations, as well as the neuroinflammation and BBB function, were not changed after social isolation. Down-regulation of the Claudin-5 has been confirmed to cause both anxiety- and depression-like behaviors during subthreshold stress (Menard et al., 2017; Dion-Albert et al., 2022). In this study, down-regulation of Claudin-5 expression in the amygdala of female mice could induced anxiety-like behaviors. In contrast, overexpression of Claudin-5 in the amygdala of female mice prevented anxiety-like behavior induced by social isolation, indicating that BBB damage in the amygdala of female mice may be a key factor of anxiety-like behaviors induced by chronic social isolation.

BBB damage and microglia activity have been shown to correlate with neuronal activation in the brain. We therefore examined neuronal activity in the amygdala of female mice after social isolation. The results showed that the number of c-fos positive cells in the BLA of female mice was significantly increased, indicating that the neurons were activated. There are as high as 80% of neurons in the BLA are glutamatergic, and the high local concentrations of excitatory neurotransmitter glutamate are associated with impaired BBB (Sharp et al., 2003; De Bock et al., 2013; Janak and Tye, 2015; Vazana et al., 2016). Previous studies have confirmed that increased levels of peripheral inflammation mediated the BBB damage, promoting the transfer of peripheral inflammatory cytokines such as IL-6 into the brain. The activation of plasma immune cells and the increase of inflammatory factors released by microglia, and the activation of microglia can regulate the firing activity of neurons, leading to activated glutamatergic neurons in the BLA and increased anxiety-like behaviors (Chen et al., 2017; Menard et al., 2017; Varatharaj and Galea, 2017; Zhu et al., 2018). These evidence supported our hypothesis that female isolated mice exhibit increased levels of neuroinflammation, impaired BBB integrity, and increased neuronal activity in the BLA, ultimately promoting anxiety-like behaviors.

The results illustrated that BBB damage in the amygdala is a crucial target in susceptibility to isolation-induced behavioral impairments in female mice. Based on previous studies, the presumed mechanism is that social isolation mediates peripheral inflammation transferring to the amygdala, activates local neuroinflammation and neurons, and induces anxiety-like behaviors. However, for this speculation, our study still has some inevitable limitations. First, the present results cannot provide strong evidence to explain the cause of the BBB damage in the amygdala of female mice, and the key mechanisms through which BBB damage induces behavioral deficits, particularly in females. In addition, we proposed that BBB damage may be caused by peripheral inflammation, neuroinflammation, or neuronal activation, and BBB damage may also play a role by promoting neuroinflammation and causing neuronal activation. Our results confirmed that Claudin-5 down-regulation in the amygdala can induce anxiety-like behaviors without social isolation. However, it also cannot explain the role of Claudin-5 in social isolation induced behavioral deficits. For the above speculation, further experiments are needed, such as whether the up- or down-regulation of the BBB affects local neuroinflammation and neuronal activation and their effects on behavioral responses to social isolation. The effects of knockdown of Claudin-5 on anxiety states under the condition of subthreshold social isolation stress should also be evaluated. Moreover, the types of activated neurons after social isolation were not determined, so we cannot provide directional guidance for further modulation of specific neuronal activity in the amygdala. Future studies aiming at characterizing distinct neurons responsible for the emotional behavior encoding will therefore be required to demonstrate how BBB alters neuronal activity by optogenetic or chemogenetic manipulations.

In conclusion, our data have revealed that female, but not male, mice exposed to chronic childhood social isolation exhibit increased anxiety-like behaviors with activated peripheral cytokines, neuroinflammation and BBB leakiness in the amygdala. Considering the crucial regulation of Claudin-5 that links BBB integrity and emotional behaviors, the development of selective regulators of tight juction proteins targeting BBB morphology and function is needed in the future particulary for individuals suffered social isolation during their early life.

## Data Availability

The original contributions presented in the study are included in the article, further inquiries can be directed to the corresponding authors.
